# Nickel as Electrocatalyst for CO_(2)_ Reduction: Effect of Temperature, Potential, Partial Pressure, and Electrolyte Composition

**DOI:** 10.1021/acscatal.4c00009

**Published:** 2024-03-08

**Authors:** Rafaël E. Vos, Marc T. M. Koper

**Affiliations:** Leiden Institute of Chemistry, Leiden University, P.O.Box 9502, 2300 RA Leiden, The Netherlands

**Keywords:** CO_2_ reduction, temperature, nickel, chain growth mechanism, coking, electrolyte effect

## Abstract

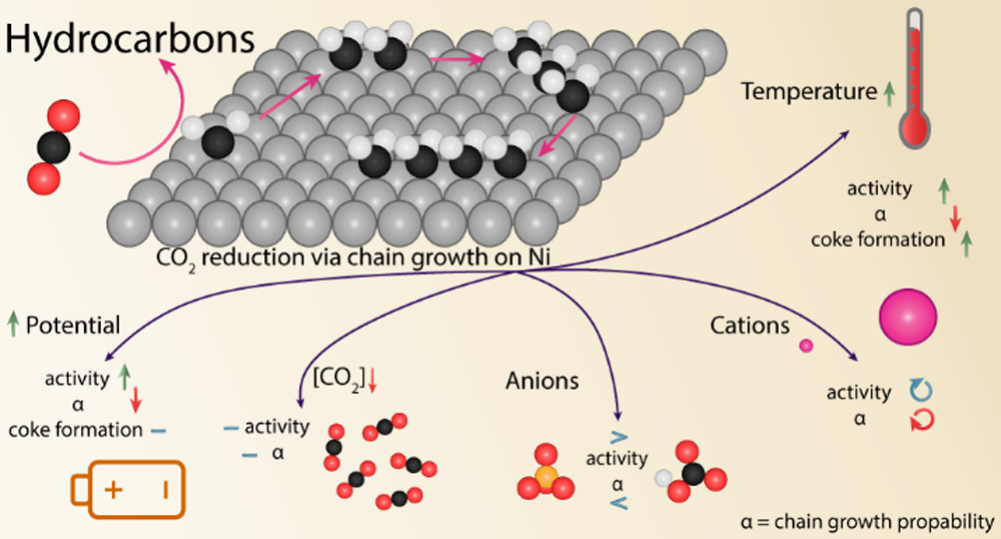

Electrochemical CO_2_ reduction on Ni has recently been shown to have the unique ability to produce longer hydrocarbon chains in small but measurable amounts. However, the effects of the many parameters of this reaction remain to be studied in more detail. Here, we have investigated the effect of temperature, bulk CO_2_ concentration, potential, the reactant, cations, and anions on the formation of hydrocarbons via a chain growth mechanism on Ni. We show that temperature increases the activity but also the formation of coke, which deactivates the catalyst. The selectivity and thus the chain growth probability is mainly affected by the potential and the electrolyte composition. Remarkably, CO reduction shows lower activity but a higher chain growth probability than CO_2_ reduction. We conclude that hydrogenation is likely to be the rate-determining step and hypothesize that this could happen either by *CO hydrogenation or by termination of the hydrocarbon chain. These insights open the way to further development and optimization of Ni for electrochemical CO_2_ reduction.

## 1 Introduction

Electrochemical CO_2_ reduction (CO_2_RR) using renewable electricity is an interesting process to produce sustainable hydrocarbons in the future. Multicarbon products are of particular relevance, as they are required in chemicals and fuels. Although several metal catalysts are able to reduce CO_2_ to useful products, Cu is the only metal able to produce C2+ products in significant amounts.^1−3^ However, the largest molecule that Cu is able to form electrochemically from CO_2_ is propanol, a C3 molecule.^4^ Efforts to produce longer hydrocarbons using Cu-derived catalysts have not been very successful up to now.^5−7^

Already in the 1990s, Kudo et al.^8^ showed that at elevated temperatures and pressures, nickel electrodes are able to reduce CO_2_ and to form longer hydrocarbons, following the Anderson–Schultz–Flory distribution known from thermochemical Fischer–Tropsch synthesis.^9^ However, the selectivities were extremely small. Recently, Zhou et al.^5^ showed that higher hydrocarbon selectivities, of up to 15%, are possible on nickel oxygenate-derived catalysts. They found linear and branched hydrocarbons with chains of up to six carbon atoms and indicated that Ni follows a Fischer–Tropsch like mechanism where CH_*x*_ intermediates are inserted into the growing hydrocarbon chain.

In the literature, it has been suggested that elevated temperatures could be beneficial for the formation of longer hydrocarbon chains.^8,10^ However, these results are convoluted by a pressure effect, as both are increased simultaneously. We have shown in previous work that temperature can play a major role in CO_2_ reduction and can influence both the selectivity and the activity. On copper, higher temperatures lead to more C2+ products, although there is an optimum around 48 °C.^11^ Ni binds the intermediate CO too strong to obtain high conversion rates of CO_2_RR,^3,12^ however an increase in temperature could help overcome this barrier and facilitate CO_2_RR. Moreover, the thermocatalytic Fischer–Tropsch reaction is influenced by temperature as well, with higher temperatures leading to shorter hydrocarbons and higher conversions.^13^ It is interesting to compare the effect of temperature on Ni to what is known about Cu, as Ni presumably follows an entirely different mechanism. Moreover, more insights are needed to understand why some catalysts like Ni, but also PdAu,^14^ Au/Ti,^15^ and Fe phthalocyanine catalysts^16^ follow a kind of chain growth mechanism, while the most commonly studied catalysts, such as Cu, Ag, Au, and Sn do not. Additionally, studying this electrochemical Fischer–Tropsch mechanism could give us more insight into other CO_2_RR mechanisms that might open up at elevated pressures and temperatures.

In this study, we investigate a nickel-derived catalyst for electrochemical CO_2_ reduction. Specifically, we investigate the effect of temperature on the performance of nickel for CO_2_RR, but also the effects of partial pressure, electrode potential, and electrolyte composition. We show that the chain growth probability does not depend intrinsically on the temperature and partial pressure, although temperature does influence the degradation rate. Electrode potential, and especially electrolyte cations, influence the chain growth probability. Moreover, CO and CO_2_ as reactants show interesting differences in activity and selectivity. Furthermore, we discuss that the rate-determining step is most likely a hydrogenation step and offer several possibilities.

## 2 Experimental Section

### 2.1 Chemicals

The electrolytes for electrolysis were prepared from Li_2_CO_3_ (99.999%, Acros Organics), NaHCO_3_ (99.7%, Sigma-Aldrich), KHCO_3_ (99.95%, Sigma-Aldrich), CsHCO_3_ (99.99%, Alfa Aesar), KH_2_PO_4_ (99.9%, Sigma-Aldrich), K_2_HPO_4_ (TraceSELECT, 99.999%, Sigma-Aldrich), and Milli-Q water (≥18.2 MΩ cm, TOC < 5 ppb). The HCO_3_^–^ electrolytes were stored with Chelex (100 sodium form, Sigma-Aldrich) to clean the electrolyte from any metal impurities.^17^ Ni(NO_3_)_2_·6H_2_O (99.999%, Sigma-Aldrich), Na_2_HPO_4_·2H_2_O (99.5%, Merck), ethanol (puriss, Honeywell), and Milli-Q water were used to prepare the deposition electrolyte. H_2_SO_4_ (95–98%, Sigma-Aldrich), H_2_O_2_ (35%, Merck), and KMnO_4_ (99%, Sigma-Aldrich) were used to clean the cells. Ar (5.0 purity, Linde), CO (4.7 purity, Linde), and CO_2_ (4.5 purity, Linde) were used for purging the electrolytes.

### 2.2 General Electrochemical Methods

The experiments were performed in a homemade PEEK H-cell or a borosilicate glass cell. The cells were cleaned prior to experiment by storing them overnight in a permanganate solution (0.2 M H_2_SO_4_, 1 g/L KMnO_4_). Before use, the cells were rinsed and submerged in diluted piranha to remove any trace of MnO_4_ and MnO_2_. Thereafter, they were rinsed again and boiled three times with Milli-Q water. A three-electrode configuration was used during experiments. The reference electrode was a commercial RHE (mini Hydroflex, Gaskatel) and was placed in the same compartment as the working electrode. An anion exchange membrane (AMVN Selemion, AGC) was used to separate the counter electrode (DSA, Magneto) from the working electrode. All the electrochemical measurements were carried out using an IviumStat potentiostat (Ivium Technologies). The flow of CO_2_ or CO (and Ar for the partial pressure experiments) was controlled using a mass flow controller (SLA5850, Brooks Instrument).

### 2.3 Electrode Preparation

The polycrystalline Ni working electrode (99.99%, Mateck) was mechanically polished with decreasing diamond polishing suspension (3.0, 1.0, and 0.25 um, Buehler) on micropolishing cloths (8 in.) until the surface was mirror polished. Then, the electrode was successively sonicated in ethanol and Milli-Q water for 3 min to remove any impurities and dried with pressurized air. Thereafter, the electrode was electrochemically polished in a 50/50 solution of H_2_SO_4_ and H_3_PO_4_ (85%, Suprapure, Merck) by applying +2 V versus a graphite counter electrode for 20 s. Subsequently, the electrode was rinsed with Milli-Q water, dried with pressurized air, and used for deposition. The Ni electrode was put in contact with the deposition solution (63 mM Ni(NO_3_)_2_ + 8 mM Na_2_HPO_4_ in Milli-Q-water with 25% ethanol) using a meniscus. Deposition was performed by cyclic voltammetry from −0.75 to 0.65 V vs RHE for 5 cycles at 15 mV/s. Next, the electrode was thoroughly rinsed with Milli-Q water and dried with pressurized air, after which it was ready to use for the electrolysis experiments. For the control experiments, the electrode was used after the electropolishing step without deposition.

### 2.4 Electrolysis Experiments

The electrolysis experiments were performed in the homemade PEEK H-cell containing 6.8 mL of 0.1 M HCO_3_^–^ electrolyte in each compartment. The PEEK H-cell was embedded in a homemade jacket which was connected to a water bath (Ecoline e100, Lauda) to control the temperature in the cell. Before electrolysis, CO_2_ (or CO) was purged through the electrolyte for 15 min while the potential at −0.1 V vs RHE was controlled to saturate the electrolyte and get the electrolyte to the proper temperature. Then, the ohmic drop was determined by electrochemical impedance spectroscopy (EIS) at −0.1 V vs RHE, and 85% ohmic drop compensation was performed for all chronoamperometry measurements. Chronoamperometry was performed for 32 min, and CO_2_ was constantly purged at 20 mL/min. At 5, 19, and 32 min, a gas sample was analyzed online using a Shimadzu 2014 gas chromatograph containing two detectors (one TCD with a Shincarbon column and one FID with a RTX-1 column). These time intervals were chosen based on the limitations of the GC. A liquid sample was taken at the end of the electrolysis. The liquid products were analyzed using high performance liquid chromatography (HPLC, Shimadzu) with a Aminex HPX-87H column (Biorad).

### 2.5 Chain Growth Probability Calculation

If the formation of hydrocarbons occurs though the polymerization of C1 intermediates, this mechanism resembles the Fischer–Tropsch synthesis mechanism.^9,18^ This mechanism follows the Anderson–Schultz–Flory model:^19^
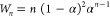
1where *W*_*n*_ is the weight percent of a product containing *n* carbon atoms and α is the chain growth probability. Eq 1 can be used to calculated the chain growth probability by plotting ln(*W*_*n*_/*n*) vs *n*:
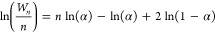
2

### 2.6 Deactivation Ratio

To get better insight in the deactivation of the catalyst over time, we compare the chain growth probability at the last and first measurement and have defined a deactivation ratio with the following expression:
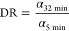
3where DR is the deactivation ratio and α is the chain growth probability. 32 min has been used due to the sampling time of the GC.

### 2.7 Partial Pressure Experiments

With the use of flow controllers, the partial pressure of CO_2_ can be altered by mixing the inlet flow with Ar gas. This allows us to change the CO_2_ concentration in the bulk electrolyte independently of the temperature. We estimate the CO_2_ concentration using Henry’s law in combination with an empirical equation to estimate Henry’s constant.^20^
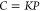
4
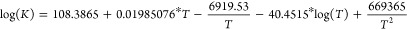
5where *C* is the concentration, *K* is the Henry’s constant, *P* is the partial pressure, and *T* is temperature.

### 2.8 Characterization of Morphology and Chemical Composition

Micrographs of the deposited Ni electrodes were obtained by scanning electron microscopy (SEM) in an Apreo SEM (ThermoFisher Scientific) with an acceleration voltage of 15 kV and an electron beam current of 1.6 nA. The chemical composition of the electrode was investigated by energy dispersive X-ray spectroscopy (EDX) using an Oxford Instruments X-MaxN 150 Silicon Drift detector coupled to the Apreo SEM. EDX data was processed with the Pathfinder X-ray Microanalysis software v1.3. The quantification of chemical elements was performed in automatic mode.

### 2.9 Raman Spectroscopy

Raman spectroscopy measurements were carried out to determine the formation of coke during the electrochemical CO_2_ reduction on Ni. The measurements were carried out ex situ both before and after the reaction. A confocal spectrometer (Witec Alpha300 R) was used with a 457 nm excitation wavelength laser. A 100 times magnification objective was used for spectra collection. The laser power was kept below 2 mW to prevent sample damage. All measurements were performed under ambient conditions at room temperature. Optical images of the electrode were recorded using the optical camera equipped with the Raman microscope setup.

## 3 Results and Discussion

### 3.1 Temperature Effect and Deactivation

Inspired by Zhou et al.,^5^ we have used a phosphate-derived Ni catalyst in this study as it is more active than metallic Ni. The phosphate-derived Ni was prepared by electrodeposition with a Ni foil as a substrate to avoid any influence of other metals in the catalyst. Figure S1 shows an SEM picture and an EDX map of the Ni electrode before electrolysis, from which it can be seen that the deposit forms a layer on top of the Ni foil. This layer does not cover the Ni foil completely but consists of small patches with cracks between where the underlaying Ni foil is still visible. The observation of cracks might be due to drying of the layer when the electrode was transferred from the electrolyte to the SEM. The deposited layer consists of Ni with oxygen and phosphor atoms, possibly forming a nickel phosphate layer mixed with nickel oxide. The atomic ratio of the EDX analysis suggests that the layer is not fully nickel phosphate, as the oxygen phosphor ratio is 8:1 instead of 4:1. However, it could also be that the layer is nickel phosphate, and the Ni underneath is oxidized, resulting in this ratio. During electrolysis, the surface changes significantly as there is not a clear layer visible anymore, but rather oxide particles on the surface are observed (Figure S2). However, phosphor is still observed in the EDX spectrum, and for the activity, the formation of the deposited precatalyst is important. Zhou et al.^5^ suggested that it is imperative that the nickel is not fully reduced during CO_2_RR to obtain moderate CO binding sites, and they reported that a nickel phosphate precatalyst works best. We also observe that the deposited Ni is a better catalyst toward CO_2_RR than the bare Ni foil (Figures S3 and S5), which is not due to a roughness effect as shown in the capacitive measurements in Figure S4, but probably due to similar effects as the catalysts of Zhou et al.^5^ Throughout the remainder of this study, the deposited Ni is used as the catalyst as it is a better catalyst and the production of hydrocarbons is sufficiently high to accurately determine parameters such as the Faradaic efficiency (FE) and chain growth probability (α) under all conditions used.

**Figure 1:**
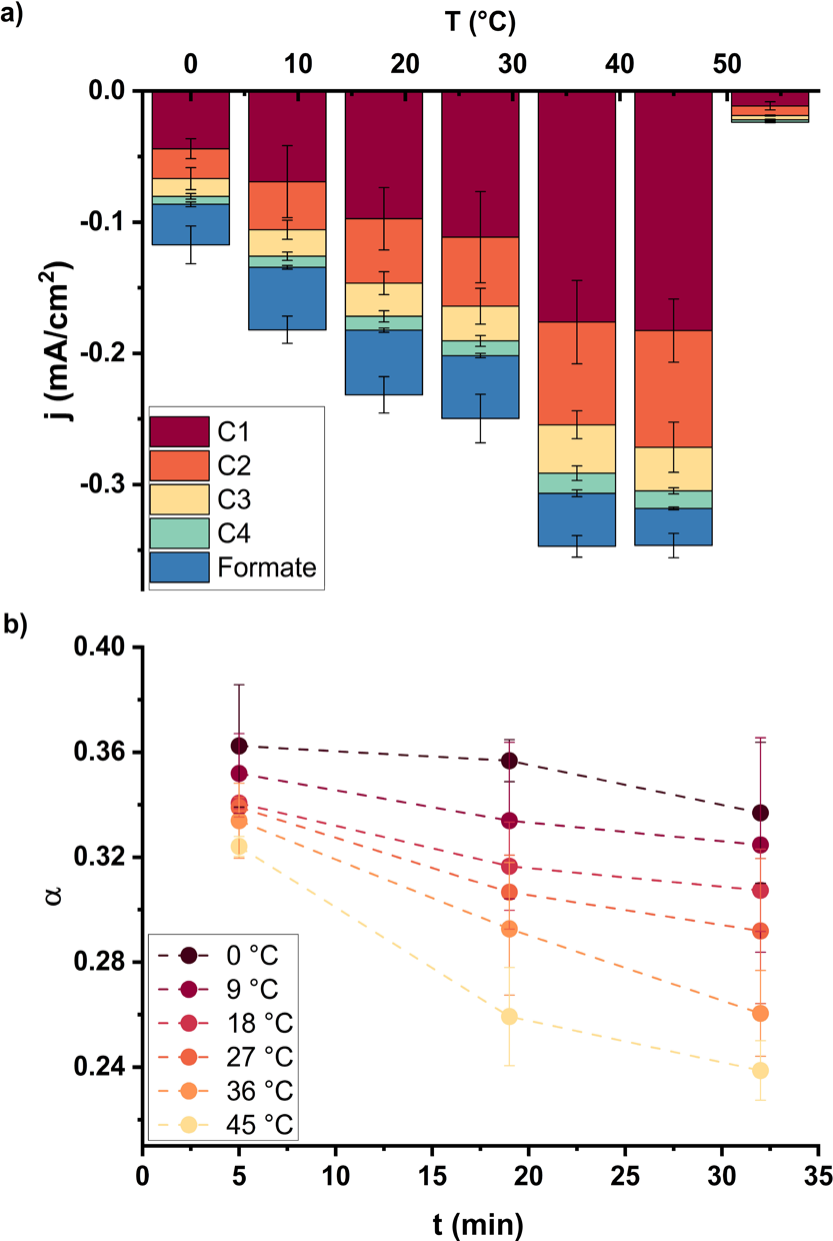
(a) Activity and (b) chain growth probability as a function of temperature on Ni in 0.1 M KHCO_3_ at −1.175 V vs RHE.

Figure 1a shows the partial current densities for the reduction of CO_2_ to different products (up to C4) on the nickel catalyst as a function of temperature. The C1 product is methane, and ethylene and ethane are the C2 products observed. Interestingly, we only detect propene as C3 product, while butene and butane are the observed C4 products. Some C5 products, namely pentene and pentane, were also detected, although in such small quantities that accurate quantification was not possible, so these products are omitted from all figures shown. Figure 1 shows that by increasing temperature, the activity toward CO_2_RR increases up to 45 °C, after which it rapidly decreases. The mechanism of CO_2_RR on Ni must be different than on Cu, which does not show the formation of C3–C5 hydrocarbons. Nickel follows a chain growth mechanism comparable with the mechanism of the Fischer–Tropsch reaction in thermocatalysis. Just like for the Fischer–Tropsch reaction, an Anderson-Flury distribution is followed, and a chain growth probability α can be determined (Figure S6). However, an interesting observation is that besides the hydrocarbons also formate is produced, for which the activity is highest between 10 and 30 °C. Kudo et al.^8^ also showed the formation of formate, especially at elevated pressures, whereas Zhou et al.^5^ did not report any formation of formate. Formate does not feature in the chain-growing Fischer–Tropsch mechanism and is likely to be formed via a different mechanism, which occurs in parallel. Figure S7 shows the FE values for the chain growth products with temperature and time. Although the FE for the CO_2_RR is low, a total FE of around 100% is reached as the hydrogen evolution reaction (HER) clearly dominates the reaction. This is generally known in literature,^3,12^ and to use Ni as an active catalyst for CO_2_ reduction, significant progress to reduce HER is still required. However, to study the chain growth mechanism on Ni at a fundamental level, these activities and selectivities are sufficient. At lower temperatures, some time is necessary to activate the catalyst, as the selectivity increases in time. At higher temperatures, the opposite occurs, and the selectivity slightly decreases over time.

The effect of the temperature on the chain growth probability is shown in Figure 1b. The chain growth probability decreases with increasing temperature, especially at longer reaction times. The decrease in chain-growth probability is the most significant at the highest temperature, as illustrated by the deactivation ratios in Figure S7. When the graphs would be extrapolated to 0 min of reaction time, it seems that the chain growth probability could be similar between the different temperatures. This would indicate that the temperature does not have an intrinsic effect on the mechanism of chain growth on Ni. However, higher temperatures lead to higher activities of CO_2_ reduction, which is accompanied by a higher deactivation rate, affecting the chain growth probability.

**Figure 2:**
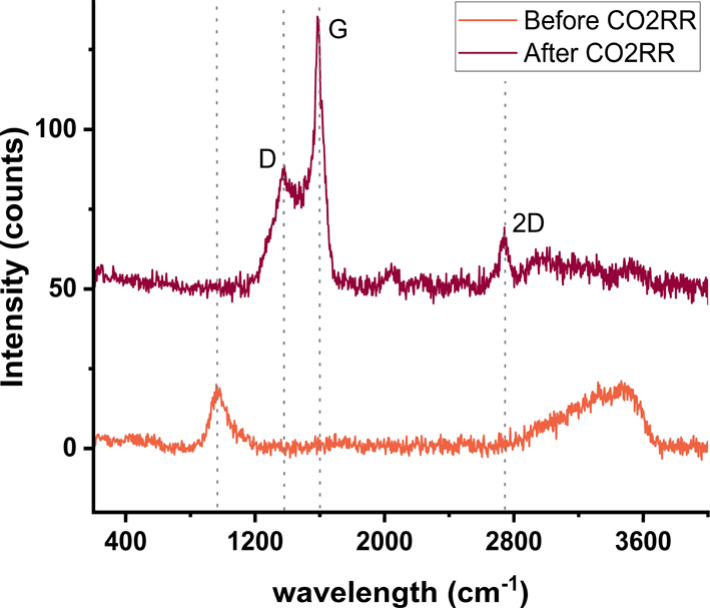
Raman spectrum before and after CO_2_RR on Ni in 0.1 M KHCO_3_ at −1.175 V vs RHE showing the formation of coke on the electrode.

Moreover, at elevated temperatures, the current increases over time, as can be observed in Figure S9a. This increase in current is due to an increase in hydrogen activity and is not caused by the catalyst preparation, as is also observed on the plain Ni electrode (Figure S9b). The formation of carbon deposits, hereafter called coking, on the electrode surface is the most probable cause of the deactivation of the catalyst, leading to a lower chain growth probability and an apparently higher HER activity. Coking is generally observed in the thermocatalytic Fischer–Tropsch reaction^21−25^ and as CO_2_RR on Ni presumably follows a similar reaction mechanism, coke is expected to form here as well. Moreover, the electrode becomes visibly black after CO_2_RR, but not after HER (Figure S10). A microscopic image shows black patterns all over the electrode after the CO_2_RR (Figure S11). To provide solid evidence for the formation of coke, Figure 2 shows Raman spectra before and after CO_2_ reduction. The peak around 960 cm^–1^ in the spectrum before CO_2_RR is likely from PO_4_.^26^ After CO_2_ reduction, intense bands are observed at 1370 and 1585 cm^–1^, which are characteristic for coke formation.^21−23,25,27,28^ The band at 1370 cm^–1^, commonly called the D band, is assigned to a more disordered type of carbonaceous species and arises due to the breakdown of the selection rule due to defects and disorder in the crystal structure.^21,27,28^ The band at 1585 cm^–1^ is called the G band and is assigned to a more graphite-like carbon.^21,22^ The 2D peak is typical for graphene,^29^ but can also be observed in graphite, where it shifts to the higher wavelengths observed here.^30^ Combined with the high intensity of the G band, this indicates that the coke formed on the Ni catalyst during electrochemical CO_2_ reduction mostly consists of a graphite-like structure. This graphitic carbon is often considered to be responsible for catalyst deactivation by blocking the active sites for CO_2_RR.^21,22,25^ The faster decrease of the chain growth probability with higher temperatures suggests that the coke could inhibit the propagation of the hydrocarbon chain, possibly by occupying adsorption sites, making it more difficult for carbon intermediates to reach the growing hydrocarbon chain. Quantification of the coke is difficult due to the inhomogeneous coke formation as can be seen from Figure S11.

### 3.2 Effect of Electrode Potential and Bulk CO_2_ Concentration

**Figure 3:**
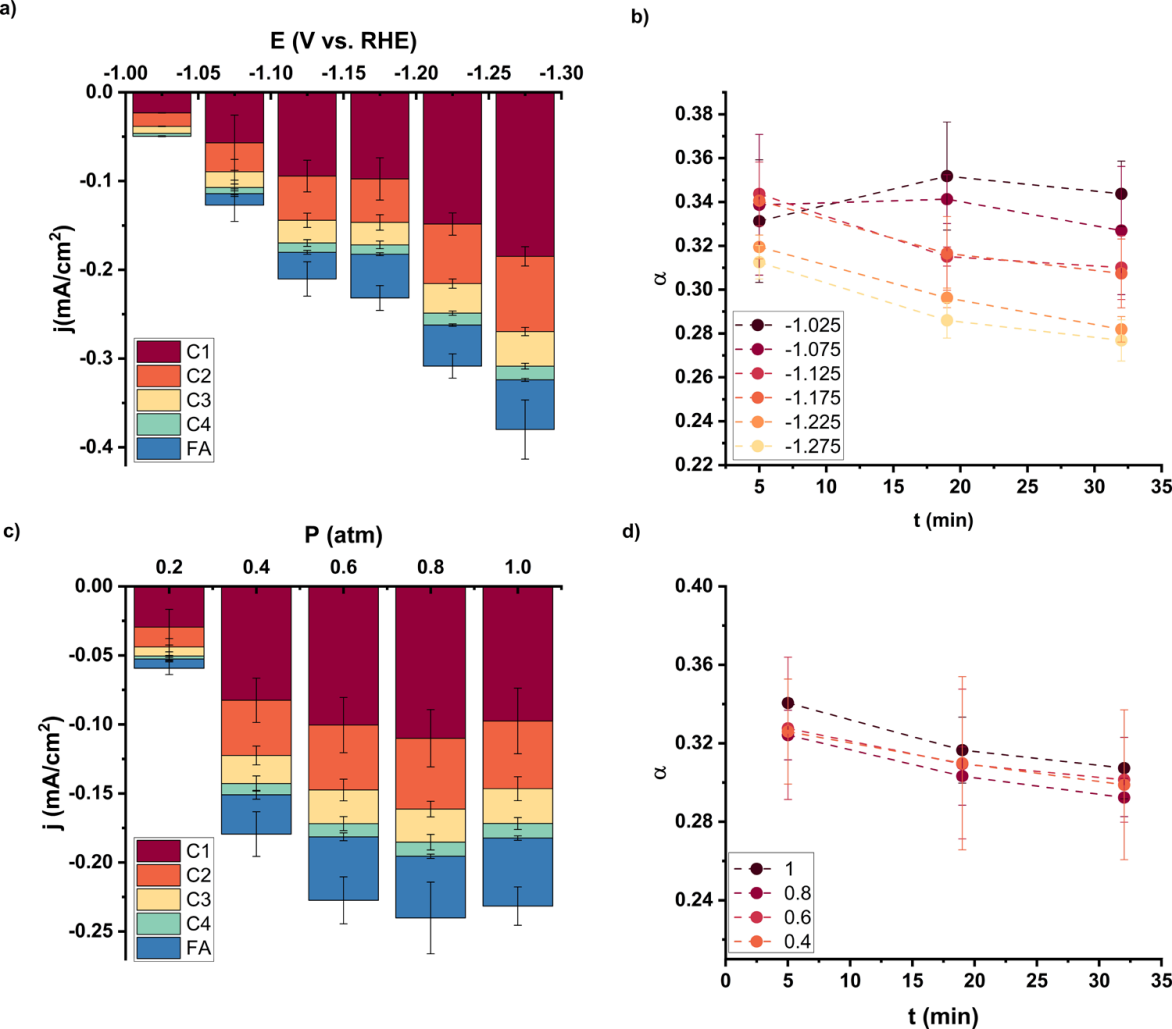
(a) Activity and (b) chain growth probability as a function of electrode potential. (c) Activity and (d) chain growth probability as a function of CO_2_ partial pressure on the Ni catalyst in 0.1 M KHCO_3_ at −1.175 V vs RHE.

To understand the effect of the temperature on the CO_2_RR on the Ni catalyst and its deactivation in more detail, additional parameters have been studied. Figure 3a,b shows the effect of electrode potential, while Figure 3c,d shows the effect of the bulk concentration of CO_2_ by adjusting the partial pressure. It can be seen that with increasing potential, the activity of CO_2_RR increases. However, the FE shows an optimum around −1.125 V vs RHE (Figure S12). Figure 3b shows that the chain growth probability is influenced by the potential, with lower overpotentials producing longer chains. Yet, at 5 min this dependency is less obvious. This time effect is not caused by deactivation as is the case with temperature. Instead, from 5 to 19 min, the chain growth probability increases at −1.075 and especially at −1.025 V vs RHE. Therefore, at the lowest potentials, time is needed to activate the catalyst, which is also apparent from the FE in Figure S11 and the deactivation ratio in Figure S8. This indicates that the Ni phosphate deposited precatalyst requires negative potential to transform into the active catalyst. We hypothesize the Ni might need to be partially reduced or the phosphate needs to be partially removed to obtain the active phosphate derived catalyst. The deactivation between 19 and 32 min seems similar at all potentials. Figure S13 shows indeed that not higher current densities but higher temperatures cause the higher deactivation rate observed in Figure 1b. In this figure, the chain growth probability of two experiments with similar current densities are compared. The experiment at higher temperature and lower potential shows higher deactivation than the experiment at lower temperature and higher potential. This is even more apparent in the deactivation ratio plotted versus both temperature and potential in Figure S7, which illustrates that there is a strong deactivation at higher temperatures. The deactivation at higher potentials stays constant, while at low potentials, the aforementioned activation is evident by a deactivation ratio higher than 1.

The bulk concentration of CO_2_ does not influence the chain growth probability or the deactivation, as can be seen in Figure 3d. The chain growth probability at 0.2 atm has not been taken into account, as the C4 production was too low to obtain an accurate value. The activity is also not impacted significantly with decreasing CO_2_ concentration until a partial pressure of 0.2 atm. Below this concentration, the activity decreases very rapidly. These results show that the effects in Figure 1 are mainly caused by the increase in temperature and not due to the changes in the bulk CO_2_ concentration.

### 3.3 Effect of Electrolyte Cation and Reactant

**Figure 4:**
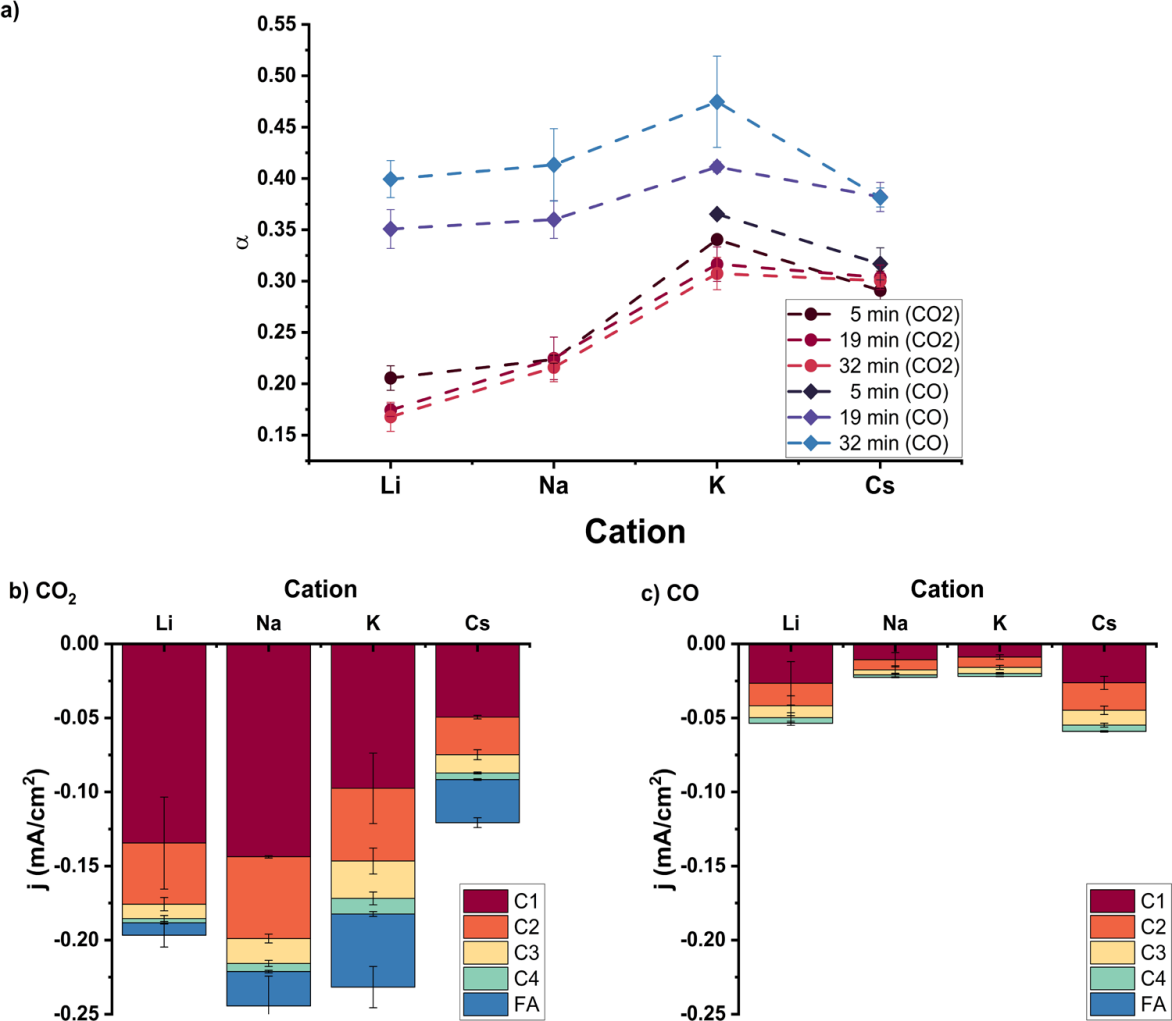
(a) Chain growth probability for different cations for both CO_2_RR and CORR, (b) activity for CO_2_RR for different cations at −1.175 V vs RHE, and (c) activity for CORR for different cations at −1.075 V vs RHE on the Ni catalyst in 0.1 M KHCO_3_.

For CO_2_ reduction, a cation effect is commonly observed.^31−34^Figure 4 shows that cations also influence the CO_2_RR and the CO reduction (CORR) on the Ni catalyst. The chain growth probability of the CO_2_RR depends significantly on the cation used and is the highest for K^+^. However, the total current density of the CO_2_RR does not depend significantly on the cation as seen in Figure 4b. Li^+^ and Na^+^ show almost similar activities as K^+^, although the product distribution is significantly altered. Cs^+^ however shows significantly lower activities, which is notable as on other metals Cs^+^ electrolytes often results in the most active catalyst.^32,33^Figure S14 shows that the deactivation rate also depends on the cation, where Cs is the most stable and Li is the most unstable. However, this does not influence the activities significantly as both at 5 min and at 32 min, the trend is similar to the time-averaged trend observed in Figure 4b.

In addition to CO_2_ reduction, we also performed experiments with CO as the reactant, as CO is expected to be a likely intermediate to form CH_*x*_ intermediates to grow the hydrocarbon chain. Figure 4a,c shows that CO reduction does indeed occur on the Ni catalyst and that the chain growth probability is higher than for CO_2_RR, although the activity is significantly lower. The chain growth probability and activity for the CORR depend significantly on time, whereas this was not the case for the CO_2_RR. It seems that the catalyst requires an activation time, as α increases with time. This might be due to the lower activities and could be comparable to the observations at lower overpotentials during the CO_2_RR in Figure 3b. Moreover, the cation effect on the CORR is significantly smaller than that for the CO_2_ reduction.

**Figure 5:**
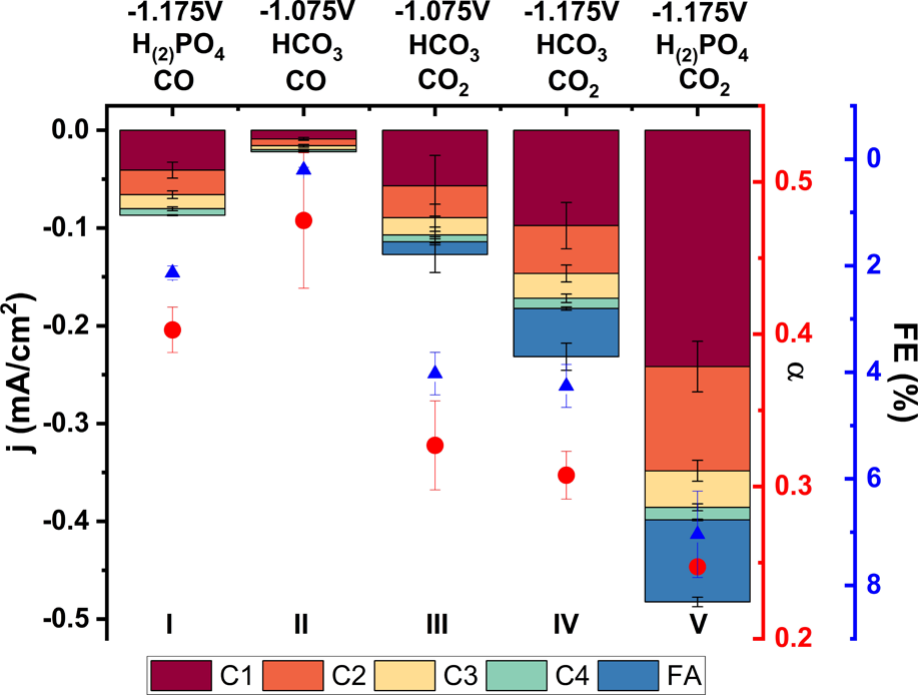
Activity, selectivity, and chain growth probability on the Ni catalyst with different applied potential, reactant, and anions. The potential given is on the RHE scale; bicarbonate and phosphate electrolytes were used at 0.1 M and either CO or CO_2_ was saturated in the solution.

To be able to compare the CO reduction vs the CO_2_ reduction in more depth, we have compared them in different electrolytes and at different electrode potentials. Experiments in Figure 4 have been performed in a bicarbonate electrolyte. However, the CO and CO_2_ saturated bicarbonate electrolytes have different pH values. If the rate-determining step (RDS) comprises a proton transfer, this can be important because both reactions should then be compared on an SHE or RHE scale. For Cu catalysts, methane and hydrogen formation have been compared on the RHE scale, while CO and the C2+ products have been compared on the SHE scale.^35,36^ To check for these pH effects, we have compared both reactions at similar potentials both at the RHE and SHE scale (Figure 5; II, III, and IV). Moreover, both reactions were performed in a pH 7 phosphate buffer, which does not change pH when saturated with either CO or CO_2_ (Figure 5; I and V). Figure 5 shows that when CO_2_RR and CORR are compared at the same potential versus SHE instead of RHE, the differences only become larger, indicating pH is important for the RDS. Moreover, the differences in activity and chain growth probability also remain when the experiments are performed in phosphate buffer. However, an interesting observation is that the activities and selectivities of both CORR and CO_2_RR increase when phosphate is used as electrolyte instead of bicarbonate. At the same time, the chain growth probabilities decrease in both cases. Phosphate is known to be a good proton donor^37^ and it might be that this increases the activity of CO_(2)_RR on Ni (also compared to HER). This would also suggest that these reactions might benefit from more acidic electrolyte conditions and that electrolyte engineering can play an important role in optimizing this reaction.

### 3.4 Discussion and Open Questions

For the thermocatalytic Fischer–Tropsch reaction, CO dissociation, oxygen removal, carbon hydrogenation, and chain termination have all been suggested as the rate-determining step and it appears difficult to reach a consensus.^18^ However, recent studies show that the hydrogenation step is the most important for the overall kinetics.^38^ For the electrochemical Fischer–Tropsch like mechanism of CO_2_RR, the CO_2_ activation is an additional step that could be rate-limiting. For CO_2_ to CO, it is generally assumed that the first electron transfer to activate CO_2_ is the RDS,^39−42^ while for C2+ formation on copper, the CO dimerization is generally considered the RDS.^43−45^ Both steps are highly sensitive to catalyst structure^46−48^ and local electrolyte composition, i.e. cation concentration and identity.^32−34^ From the data in Figure 5, we suggest that the CO_2_ dissociation is not the RDS for the formation of hydrocarbons on Ni as the activities for the CO_2_RR are higher than for the CORR. If the CO_2_ activation would be the RDS, we would expect higher activities when this step is bypassed by starting with CO as reactant. Similar activities are expected if the RDS is after the CO_2_ activation, as has been observed on Cu electrocatalysts.^49,50,51^Moreover, CO_2_ activation as an RDS would result in a dependence on the bulk CO_2_ concentration. However, Figure 3c shows that this is not the case unless very low concentrations are used.

The apparent activation energy for the different products can be obtained by an Arrhenius plot (Figure S15). This activation energy is about 60 kJ/mol and is similar for the different CO_2_RR products. This would suggest that the rate-determining step is the same step for all products. Moreover, we observe an anion effect with a better proton donor, leading to higher activities. This indicates that a hydrogenation step is the RDS. Another indication for hydrogenation as the rate-determining step is the effect of pH, as seen in Figure 5. These results indicate that a proton transfer is involved in the rate-determining step, as is the case for hydrogenation.

Equivalent to the thermocatalytic Fischer–Tropsch reaction, the termination of the hydrocarbon chain by either hydrogenation or hydrogenation of *CO could be the rate-determining step. From our data, it is not possible to exclude CO reduction as the RDS as we have done for CO_2_. If CO reduction is the RDS, we might expect that CO_2_RR and CORR give equal activities as the activation of CO_2_ to CO should not have any effect. However, we observe that CO reduction leads to activities lower than those of the CO_2_RR. This might be explained if the local CO concentration is higher during CO_2_ reduction than during CO reduction due to the low CO solubility. A dependence on the CO coverage could also explain why the activity decreases only at low CO_2_ concentrations, where the CO coverage might be more limited. However, it could also be that CO adsorbs strongly to the surface, which blocks sites for hydrogenation of the hydrocarbon chain and thus lowers the activity. This could be another explanation why CO leads to lower activities, something that also has been observed in thermocatalytic Fischer–Tropsch synthesis, where CO has a negative reaction order.^38^ Another possible explanation for the observation that CO_2_RR is more active than CORR is that they follow different pathways, as has been suggested by Zhou et al.^5^ However, this is merely an hypothesis and could also be due to different reaction sites for CO and not to a difference in mechanism, as has been observed for cofeeding experiments on Cu electrodes.^52,53^ An argument in favor of *CO hydrogenation as RDS is that for the formation of methane on Cu, the RDS is hydrogenation of *CO to form *CHO.^54^

When the chain growth probability is plotted versus activity, a negative trend is observed (Figure S16). The chain growth probability can be defined as^9^
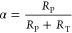
6where α is the chain growth probability, *R*_P_ is the rate of propagation, and *R*_T_ is the rate of termination. The negative trend between activity and chain probability could be related to the deactivation caused by coke formation. However, this trend is also seen for the experiments at different potentials, where no difference in deactivation due to coking has been observed (Figure S7). This negative trend might therefore indicate that the hydrogenation to terminate the chain is the rate-determining step instead of *CO hydrogenation. This would namely mean that an increase in activity is related to an increase in *R*_T_, which leads to a decrease in chain growth probability. This is similar to the thermocatalytic Fischer–Tropsch reaction, where at low pressures, the termination step is also considered rate limiting.^38^

The only observation that is more difficult to align directly with hydrogenation as the rate-determining step is the cation effect observed in Figure 4. The cation effect on its own can still be explained as the hydrogenation can be cation mediated, similar to the hydrogen evolution reaction.^55^ However, this would not explain why the cation effect is larger for CO_2_RR than for CORR. It might be that the cations only start to be important at higher coverage of intermediates and that when CO is absorbed, these coverages cannot be reached. Moreover, the cations do not follow the same negative trend between the activity and chain growth probability. This might indicate that the cations interact in a different way with chain growth on the Ni catalyst. These observations strongly suggest that hydrogenation is involved in the rate-determining step. However, whether this is the hydrogenation of *CO or the hydrogenation that leads to termination of the hydrocarbon chain cannot be answered conclusively. A systematic mechanistic study including in situ spectroscopic experiments is required to determine the exact rate-determining step and the precise role of the cations. This is outside the scope of the current study but shows that many open questions remain to be answered about the electrochemical chain growth mechanism.

## 4 Conclusions

In this work, we investigated the electrochemical reduction of CO_2_ on Ni via an electrochemical Fischer–Tropsch-like mechanism. We observe that temperature enhances the activity but also the formation of coke, which deactivates the catalyst. This results in a faster decrease of the chain growth probability with time at higher temperatures. The bulk concentration of CO_2_ hardly influenced the reaction. The chain growth probability is mainly influenced by potential and especially the electrolyte composition. Lower potentials lead to lower activities and higher chain growth probabilities. Moreover, K^+^ containing electrolytes form the longest hydrocarbons, although the effect is smaller with CO as a reactant instead of CO_2_. Also, the anions can influence the reaction, where better proton donating anions seem to increase the rate of reaction. The rate-determining step is most likely a hydrogenation step and we hypothesize this could either be the hydrogenation of *CO or the hydrogenation of the hydrocarbon chain to terminate its growth. These results open the way to the further development of Ni as a catalyst for electrochemical CO_2_ reduction. Furthermore, they give insight into the Fischer–Tropsch-like mechanism, which makes it possible to optimize this reaction on other catalysts. However, more research is needed to understand the mechanism in more detail and especially the influence of the electrolyte composition on the mechanism.

## Supporting Information Available

Additional experimental results including SEM-EDX data, an Anderson-Schultz-Flury plot, Faradaic efficiency plots, additional deactivation data including picture of the catalyst after electrolysis, Arrhenius plots and a plot relating activity and chain growth probability (DOCX)

## Abbreviations

CO_2_RR: electrochemical CO_2_ reduction
CORR: electrochemical CO reduction
FE: Faradaic efficiency
GC: gas chromatograph
HER: hydrogen evolution reaction
HPLC: high performance liquid chromatography
RHE: reversible hydrogen electrode
