# Supplementary material for: Nickel as Electrocatalyst for CO(2) Reduction: Effect of Temperature, Potential, Partial Pressure, and Electrolyte Composition
Source: ACS Catal. 2024 Mar 8;14(7):4432–40. doi: 10.1021/acscatal.4c00009 (PMC11002821; doi:10.1021/acscatal.4c00009)
Supplement: Supplementary file 1 — cs4c00009_si_001.docx [file cs4c00009_si_001.docx]

**Supporting Information**

Nickel as electrocatalyst for CO_(2)_ reduction; effect of temperature, potential, partial pressure and electrolyte composition

Rafaël E. Vos^a^, Marc T.M. Koper^a^*

^a^ Leiden Institute of Chemistry, Leiden University, P.O.Box 9502, 2300 RA Leiden, The Netherlands

*E-mail: m.koper@chem.leidenuniv.nl

**Table of content:**

**SEM-EDX images 2**

**Effect of deposition 4**

**Anderson-Schultz-Flury plot 5**

**Faradaic efficiency; temperature effect 6**

**Deactivation by coking 6**

**Faradaic efficiency; potential effect 9**

**Comparing chain growth probabilities 9**

**Cation dependance of deactivation ratio 10**

**Arrhenius plots 10**

**Relationship chain growth probability and activity 11**

**SEM-EDX images**


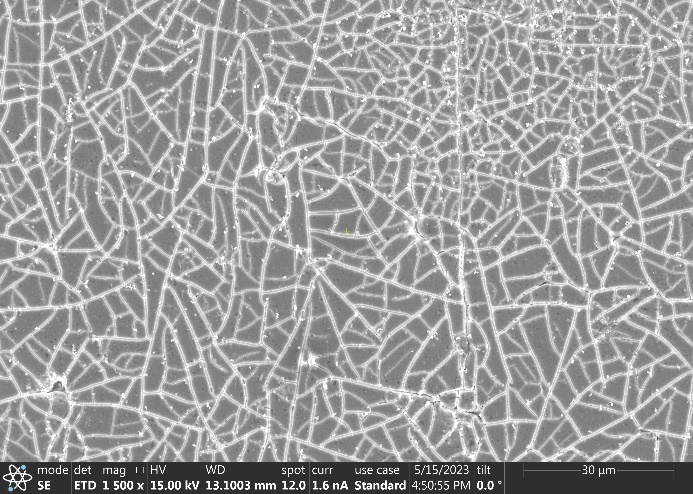

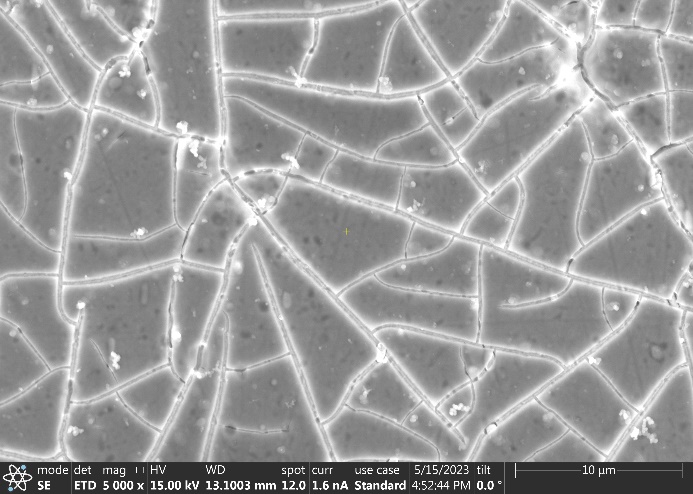










Figure S1: SEM image of the Ni deposited electrode before CO2RR at respectively a) 1500 and b) 5000 magnification. EDX maps at 50 000 magnification showing c) the SEM image d) nickel e) oxygen f) phosphor.


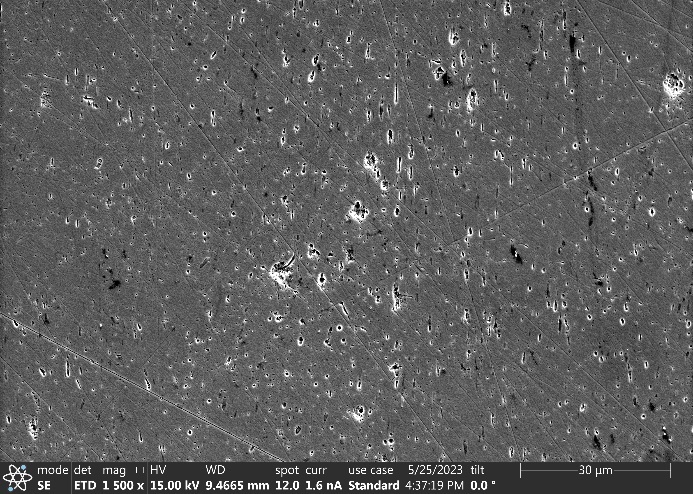

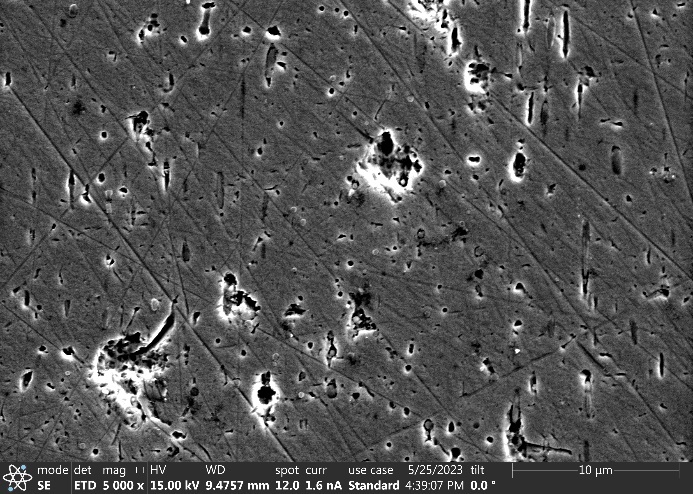










Figure S2 SEM image of the Ni deposited electrode after CO2RR for 32 minutes at respectively a) 1500 and b) 5000 magnification. EDX maps at 50 000 magnification showing c) the SEM image d) nickel e) oxygen f) phosphor.

**Effect of deposition**





Figure S3: Activity for CO_2_ reduction on a polished polycrystalline Ni electrode with and without Ni deposition. Deposition shows higher activities at -1.175 V vs RHE at 18 °C





Figure S4: a) Determination of the double layer capacitance by cyclic voltammetry. The double layer capacitance was determined at 0.075 V vs RHE and used to calculate a roughness factor (Rf). b) the roughness factor was used to normalize the deposited layer to the non-deposited catalyst, showing that the enhancement is not due to a roughness effect.





Figure S5: Chain growth probability for CO_2_ reduction on a polished polycrystalline Ni electrode with and without Ni deposition at -1.175 V vs RHE at 18 °C. Deposition shows higher chain growth probability

**Anderson-Schultz-Flury plot**





Figure S6: Example of an Anderson-Flory plot to obtain the chain growth probability on the Ni electrode

**Faradaic efficiency; temperature effect**





Figure S7: Faradaic efficiency as function of temperature on Ni in 0.1 M KHCO_3_ at -1.175 V vs RHE towards a) the total hydrocarbon formation (C1 up to C4 hydrocarbons) during CO_2_ reduction both averaged and as function of time and b) towards H_2_ and total FE

**Deactivation by coking**





Figure S8: Deactivation ratio as function of temperature and potential







**b)**

**a)**

Figure S9: a) Time dependence of current at different temperatures b) Time dependence of current at 45 °C both with the deposited electrode and the plain Ni electrode


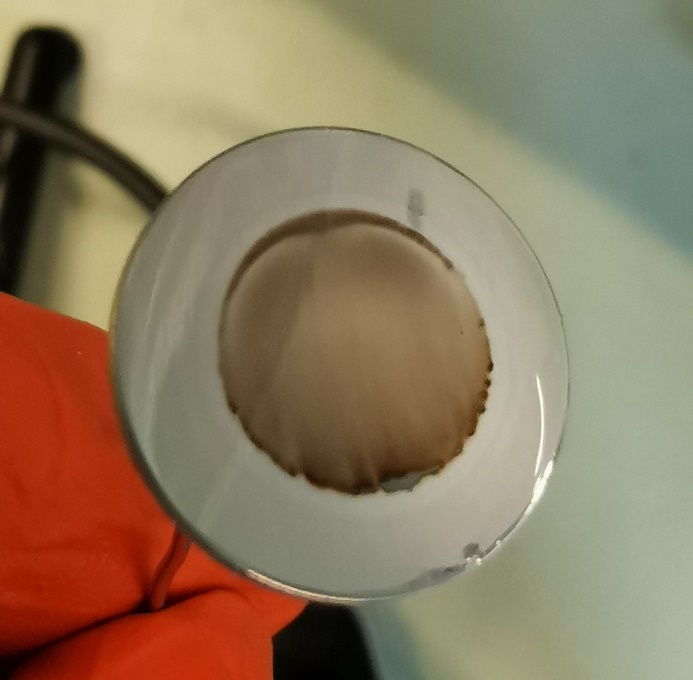

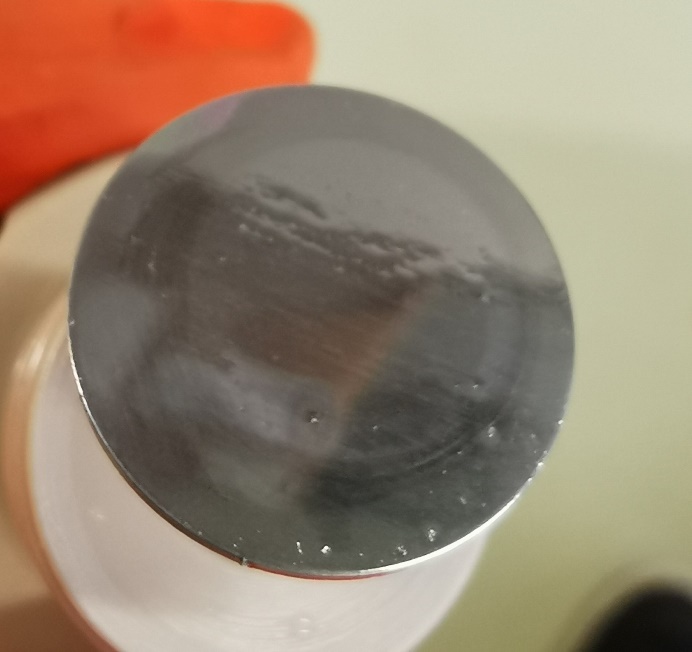


Figure S10: Photographs of the electrode after a) CO2RR and b) HER showing that the coke only forms after CO2RR


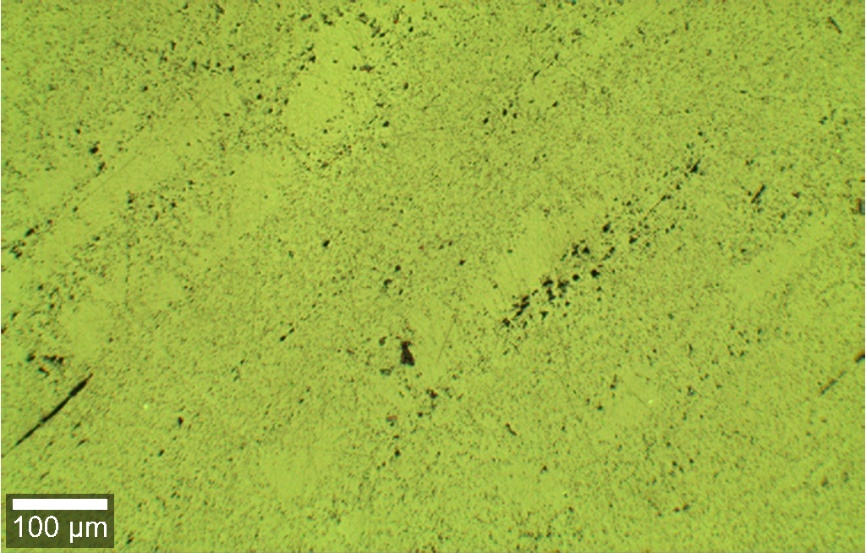


Figure S11: Microscopy picture of the electrode after CO2RR showing the black deposits on the surface.

**Faradaic efficiency; potential effect**





Figure S12: Faradaic efficiency as function of potential and time on Ni in 0.1 M KHCO_3_ at 18 °C

**Comparing chain growth probabilities**





Figure S13: Chain growth probability in time for 2 different experiments with similar activity showing that higher temperature causes deactivation of the catalyst

**Cation dependence of the deactivation ratio**

**

**

Figure S14: Deactivation ratio as function of cation identity

**Arrhenius plots**



Figure S15: a) Arrhenius plot after 5 min for methane, b) and for the other hydrocarbons which have similar activation energies

**Relationship chain growth probability and activity**





Figure S16: chain growth probability α plotted vs the activity at different conditions: a) at different potentials (data from figure 3) b) at different anions and with CO or CO_2_ as reactant (data from Figure 5) c) at different temperature (data from Figure 1) d) with different cations (data from Figure 4)
